# Development of a clinical diagnostic model for Bell’s palsy in patients with facial muscle weakness

**DOI:** 10.17305/bb.2024.10677

**Published:** 2024-12-01

**Authors:** Hongzhu Li, Guangxian Chen, Guoan Lai, Shiyu Lin, Jingchun Zeng, Liming Lu, Yuemei Li, Shuxin Wang

**Affiliations:** 1 Department of Rehabilitation Medicine, Guangzhou Eighth People’s Hospital, Guangzhou Medical University, Guangzhou, China; 2 Center of Rehabilitation, The First Affiliated Hospital of Guangzhou University of Chinese Medicine, Guangzhou, China; 3 The First Clinical Medical College of Guangzhou University of Chinese Medicine, Guangzhou, China; 4 South China Research Center for Acupuncture and Moxibustion, Medical College of Acupuncture and Moxibustion and Rehabilitation, Guangzhou University of Chinese Medicine, Guangzhou, China

**Keywords:** Bell’s palsy, facial muscle weakness, primary care, diagnostic tool, MRI, clinical indicators

## Abstract

Early diagnosis of Bell’s palsy is crucial for effective patient management in primary care settings. This study aimed to develop a simplified diagnostic tool to enhance the accuracy of identifying Bell’s palsy among patients with facial muscle weakness. Data from 240 patients were analyzed using seven potential clinical evaluation indicators. Two diagnostic benchmarks were established: one based on clinical assessment and the other incorporating magnetic resonance imaging (MRI) findings. A multivariate logistic regression model was developed based on these benchmarks, resulting in the construction of a predictive tool evaluated through latent class (LC) models. Both models retained four key clinical indicators: absence of forehead wrinkles, accumulation of food and saliva inside the mouth on the affected side, presence of vesicular rash in the ear or pharynx, and lack of pain or symptoms associated with tick exposure, rash, or joint pain. The first model demonstrated excellent discriminative ability (Area under the curve [AUC] ═ 0.96, 95% confidence interval [CI] 0.94–0.99) and calibration (*P* < 0.001), while the second model also showed good performance (AUC ═ 0.88, 95% CI 0.83–0.92) and calibration (*P* ═ 0.005). Bootstrap validation indicated no significant overfitting. The LC defined by the first model significantly aligned with the clinical diagnosis group, while the second model showed lower consistency.

## Introduction

Facial weakness is a common symptom caused by various conditions, with Bell’s palsy being one of the most prevalent reasons [1–4]. Typical manifestations include the disappearance of facial folds and nasolabial grooves, smoothing of forehead wrinkles, and drooping of the mouth corners [5, 6]. These symptoms affect the patient’s appearance and confidence and can significantly impact daily activities, such as swallowing difficulties and dry eyes [7, 8]. In primary care settings, distinguishing between idiopathic and other causes of peripheral facial paralysis in patients presenting with facial muscle weakness is crucial for treatment and prognosis, yet this task poses significant challenges for clinicians [9–11]. Faced with this scenario, physicians need to carefully consider the patient’s medical history, conduct a detailed physical examination, and may rely on specific diagnostic tests to assist in decision making [12].

The etiology of Bell’s palsy remains incompletely understood but is widely believed to be associated with viral infections, such as herpes simplex virus (HSV) and immune system abnormalities [13–15]. Although various theories exist, the exact pathophysiological mechanisms remain a focal point of research [16, 17]. This uncertainty complicates the diagnostic process, especially in the absence of specific biomarkers [18, 19]. Therefore, clinicians often rely on a detailed evaluation of medical history and physical examination to make a diagnosis [20–23]. This process aims to rule out other conditions that could lead to similar symptoms, such as stroke, Lyme disease, or brain tumors [24–26].

An accurate diagnosis is particularly crucial for potential Bell’s palsy patients as it directly affects treatment choices and prognosis [2–4]. Early recognition and treatment can significantly improve patient outcomes, while delayed diagnosis or misdiagnosis may lead to unnecessary anxiety and treatment delays [27, 28]. In some cases, if patients present with atypical symptoms like eye tremors, hearing loss, or tinnitus, magnetic resonance imaging (MRI) may be used to rule out more serious conditions like brain tumors or cerebrovascular diseases [29–32]. However, for most Bell’s palsy patients, MRI results may appear normal, highlighting the importance of relying on clinical assessment in the absence of precise biomarkers [33–35].

Despite these challenges, clinicians can still make a highly confident diagnosis of Bell’s palsy by integrating multiple indicators derived from clinical symptoms. This comprehensive assessment aims to enhance diagnostic accuracy by identifying typical and atypical features of the condition. For instance, the absence of forehead wrinkles is typically not seen in other types of facial paralysis, serving as a crucial distinguishing factor for Bell’s palsy [5, 36, 37]. Given that treatment decisions are often based on diagnostic accuracy, developing a clinical diagnostic model that incorporates multiple predictive factors is essential for guiding treatment choices and improving patient outcomes.

Given the limitations and challenges of existing diagnostic methods, this study aims to provide a diagnostic model for primary care clinicians to more accurately diagnose Bell’s palsy. By analyzing clinical data of patients with facial muscle weakness and considering whether to perform adjunctive tests like MRI, this research aims to develop a comprehensive diagnostic tool to enhance the identification and management of Bell’s palsy in primary care settings. Additionally, the study aims to explore the role and challenges of different reference standards in the diagnostic process, thereby offering clinicians more accurate and practical diagnostic approaches.

## Materials and methods

### Patient cohort

This study analyzed data from patients with facial palsy diagnosed by primary care physicians in observational cohort studies conducted at the Guangzhou University of Chinese Medicine Affiliated Hospital. Patients completed a questionnaire, underwent a standardized clinical assessment by an acupuncturist, and underwent a brain MRI scan within two weeks of assessment (unless clinically contraindicated).

At the end of the clinical assessment, the acupuncturist recorded: 1) the diagnosis of Bell’s palsy and 2) their confidence in the diagnosis (0%–100%). Clinical diagnosis was made solely based on medical history and physical examination findings, with MRI results not being part of the diagnostic process. In this study, the term “Bell’s palsy” refers specifically to facial nerve involvement. MRI scans were graded by a senior consulting neuroradiologist who had no knowledge of any clinical information about the patients, only aware of the presence of facial palsy in the patients (without specifying the specific side of the palsy). The radiologist provided a clinical report indicating the certainty, likelihood, or absence of the lesion responsible for the condition.

### Research findings and diagnostic criteria

The primary focus of this study is the diagnosis of Bell’s palsy. To establish a diagnostic model, we selected two different reference standards:

#### Model (i): Clinically diagnosed Bell’s palsy with high confidence (≥ 80%)

This standard was employed when the attending clinician documented facial paralysis due to peripheral facial neuritis and had at least 80% confidence in the diagnosis, thereby qualifying it as Bell’s palsy. The choice of 80% or higher diagnostic confidence as a threshold was based on the significant increase in reliability among clinicians diagnosing facial paralysis using this criterion.

#### Model (ii): Clinically diagnosed Bell’s palsy with high confidence (≥ 80%), with no lesion found on MRI

The second reference standard combines the clinical diagnosis by physicians with MRI findings indicating no, possible, or definite responsible lesions. Relying solely on clinical diagnosis may introduce bias, as the evaluation of predictive factors can be influenced by the information already known to the physician, making it less objective.

By setting these two reference standards, this study aims to provide a more comprehensive and objective assessment of Bell’s palsy diagnosis while considering the limitations of relying solely on clinical diagnosis without MRI support. This approach helps improve diagnostic accuracy and provides a scientific basis for the early diagnosis and treatment of facial paralysis in patients.

### Predictor selection and sample size determination

Six potential predictors were selected for the diagnostic model based on expert consensus and prior research findings from a plethora of self-report and clinical evaluation data. The selection of these predictors was grounded on two primary rationales: first, clinical assessment items crucial for distinguishing Bell’s palsy were identified by experts using the Delphi method; second, items previously validated in a multivariate diagnostic model to effectively identify facial paralysis conditions [38]. Due to low-frequency numerical issues in the data, logistic regression analysis of individual variables became impractical, leading to the final determination of six predictors for the multivariable model analysis.

This study utilized secondary data from existing randomized controlled trials to conduct the diagnostic model analysis, thus obviating the need for new sample size calculations. In line with existing guidelines, which stipulate a minimum of ten events per predictive factor [39], and considering that there were 100 patients in the smallest outcome category (i.e., patients diagnosed with reference pain in model (i)), the sample size was deemed adequate. This approach aimed to develop both an accurate and practical diagnostic model, intended to enhance the accuracy and reliability of Bell’s palsy diagnosis in primary care settings.

### Design and sensitivity analysis of a simplified scoring tool

In order to derive a simplified scoring tool from the best-performing model, this tool aims to provide the probability of Bell's palsy, for patients with facial muscle weakness. Each regression coefficient of the predictive factors in the final model was divided by the smallest coefficient to convert each coefficient to an integer [40]. Subsequently, these scores and their corresponding probability of outcomes were presented in parallel [41]. This approach streamlines the traditional scoring system, enabling clinicians to assess the likelihood of a patient having a specific disease in a more intuitive manner and thus providing more precise medical guidance to patients.

To validate the robustness of the model, we conducted a multivariable logistic regression analysis using only MRI results as a reference standard, comparing it with models in the literature that solely rely on MRI findings as the reference standard. Log odds ratios (log ORs), corresponding CIs, and the area under the ROC curve (AUC) of this additional model were compared with the original two models. Through this sensitivity analysis, we could evaluate the changes in the predictive ability of the model when relying only on MRI results as a reference standard, further confirming the accuracy and applicability of the model.

### Ethical statement

All participants in this study provided written consent to participate, under the supervision of trained research physicians from Guangzhou University of Chinese Medicine Affiliated Hospital, by signing an informed consent form. All procedures, including the consent process, were approved by the Ethics Committee of Guangzhou University of Chinese Medicine Affiliated Hospital (K-2022-124).

### Statistical analysis methods and internal validity assessment

The relationship between each predictive factor and the diagnosis of Bell’s palsy was initially investigated through univariate logistic regression analysis, based on the two predefined reference standards. Subsequently, we employed multivariate logistic regression analysis and utilized backward stepwise selection (with a significance level set at 0.05) to identify the predictive factors included in the final model. Nearly all baseline predictive factors had no missing data, allowing for a complete case analysis. The impact of each predictive factor in the final model was presented in terms of beta coefficients and ORs with 95% CIs. Calibration and discrimination measurements were conducted to assess the predictive performance of the model. Calibration was assessed by plotting Lowess smoothed curves showing the observed outcomes against the probabilities predicted by the logistic regression model [42], with perfect calibration indicated by a slope of a 45∘ line; deviation from this line signals inadequate calibration. Additionally, calibration assessment was supplemented by the Hosmer–Lemeshow goodness-of-fit test [43], with a *P* value ≥ 0.05 denoting a well-fitting model. The model’s discrimination, i.e., the ability to distinguish between the presence or absence of Bell’s palsy diagnosis, was summarized by the area under the ROC, with AUC values ranging from 0.5 (no discrimination ability) to 1.0 (perfect discrimination ability) [44].

Further, 1000 bias-corrected bootstrap samples were utilized to evaluate the internal validity of the final model, and adjusted AUC values were computed to reflect the discriminative performance of the model validated internally. Considering potential imperfections in the reference standards, we employed probability statistical methods utilizing latent class (LC) modeling [45]. This approach allowed us to model response probabilities of clinical assessment items without the knowledge of patients’ true classifications (diagnosis) [46]. Through this technique, patients’ latent or underlying groups could be identified based on the response of clinical assessment items, obviating the need for traditional reference standards. Each patient was reclassified based on the LC model of the two solutions, assuming the two LCs correspond to groups of patients with and without the target condition. LC modeling was conducted using MPlus v5, while SPSS v22 was employed for diagnostic modeling and descriptive analysis. All statistical analyses were performed using the R software version 3.4.3 (www.R-project.org) and Empower version 4.2.8 (www.empowerstats.com, X&Y Solutions, Inc.). Throughout the analyses, a two-tailed *P* value < 0.05 was considered statistically significant.

## Results

### Comparative analysis of participant characteristics in Bell’s palsy diagnostic model development

**Figure 1.**
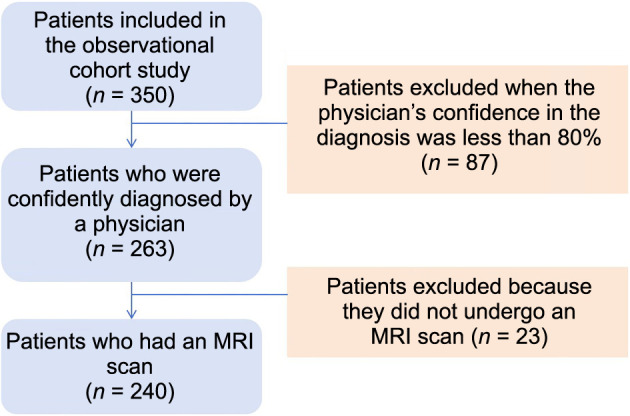
**Flowchart of inclusion and exclusion criteria for the diagnosis model research of patients with Bell’s palsy.** MRI: Magnetic resonance imaging.

This study aimed to develop and evaluate a diagnostic model for patients with Bell’s palsy, while also exploring participants’ clinical characteristics through LC modeling. A total of 350 patients with facial weakness presented for medical care, with 240 included in the development analysis of the diagnostic model and construction of the LC model. The exclusion criteria (Figure 1) were as follows: (1) physician diagnostic confidence below 80% (*n* ═ 87) and (2) patients not undergoing MRI scans (*n* ═ 23).

Table 1 displays the characteristics of patients included in the diagnostic model sample (*n* ═ 240) and those excluded from model construction analysis (*n* ═ 110). The patients in the diagnostic model development group had a higher proportion of individuals above 65 years old (15% vs 10%), a lower proportion of females (40% vs 51%), and a higher percentage of having two or more comorbidities (40% vs 32%). In terms of clinical features, the patients in the diagnostic model group showed higher proportions in aspects, such as facial folds and nasolabial groove disappearance (84.2% vs 78.2%), forehead wrinkling (75.4% vs 68.2%), food and saliva accumulation in the affected side of the mouth (81.7% vs 57.3%), and retention of affected side facial sensation (82.1% vs 59.1%) compared to patients not included in the model analysis.

**Table 1:** Characteristics of patients eligible and ineligible for diagnostic model development based on Bell’s palsy data

**Characteristics**	**Diagnostic model cases (*n* ═ 240)**	**Excluded cases (*n* ═ 110)**
Age (years), mean (± SD)	48.2 (± 16.2)	44.84 (± 14.9)
*Age categories*		
18–34 years	58 (24.17)	38 (34.55)
35–44 years	41 (17.08)	19 (17.27)
45–54 years	56 (23.33)	22 (20)
55–64 years	48 (20)	20 (18.18)
65+ years	37 (15.42)	11 (10)
*Sex*		
Female	95 (39.58)	56 (50.91)
Male	145 (60.42)	54 (49.09)
Obese BMI category*	11 (4.58)	6 (5.45)
Acute onset	240 (100)	110 (100)
Monolateral onset	235 (97.9)	107 (97.3)
The facial crease and nasolabial fold disappear	202 (84.2)	86 (78.2)
The forehead unfurrows	181 (75.4)	75 (68.2)
Food and saliva pool in the affected side of mouth	196 (81.7)	63 (57.3)
Facial sensation is preserved on the affected side	197 (82.1)	65 (59.1)
Vesicular eruption in ear canal or pharynx without pronounced prodrome of pain	215 (89.6)	78 (70.9)
Denial history of tick exposure, rash, or arthralgias	184 (76.7)	62 (56.4)
MRI scan without responsible lesions	148 (61.7)	36 (32.7)
Two or more other health problems^**^	95 (39.6)	35 (31.8)
Fair/poor general health	174 (72.5)	86 (78.2)
Clinical diagnosis of Bell’s palsy	156 (65.0)	48 (43.6)

All the numbers represent frequencies (percentages) unless stated otherwise as mean (SD). *Obese BMI category ═ 30–40+ kg/m^2^. **The comorbidity health problems included chest problems, heart problems, raised blood pressure, diabetes, circulation problems in the legs. SD: Standard deviation; BMI: Body mass index; MRI: Magnetic resonance imaging.

During the research on the development of Bell’s palsy diagnostic model and feature analysis, we observed significant differences in clinical characteristics among the participant groups. Furthermore, our analysis revealed specific features of facial paralysis patients in the diagnostic model development and LC model construction, findings that may hold important implications for future diagnostic and treatment strategies.

### Univariate diagnostic model analysis and prediction

In this section of the study, we developed and validated diagnostic models for 240 patients with Bell’s palsy. Utilizing Model (i) as a reference standard, based on a high confidence level (≥ 80%) of clinical diagnosis, 65% (*n* ═ 156) of patients were diagnosed with Bell’s palsy. Applying Model (ii) as a reference standard, in conjunction with MRI results and high-confidence clinical diagnosis, 58% (*n* ═ 140) of patients were diagnosed with Bell’s palsy.

**Figure 2.**
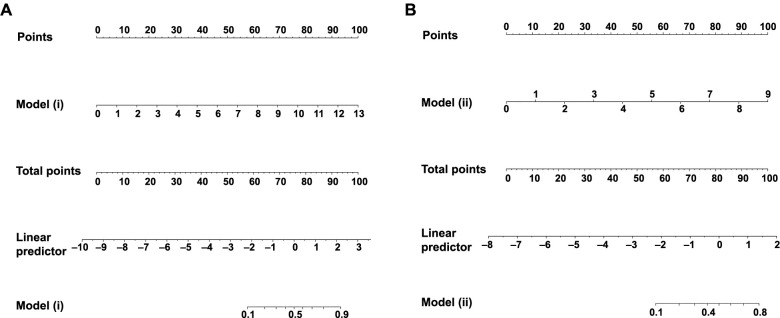
**Allocation of points and linear predictive values in the diagnostic model for Bell’s palsy.** This figure illustrates the scores and predicted probabilities of two univariate diagnostic models for Bell’s palsy. (A) Model (i) based on high-confidence clinical diagnosis (≥ 80%); (B) Model (ii) incorporating high-confidence clinical diagnosis combined with MRI results. The point allocation for each model is based on relevant clinical variables of 240 patients. MRI: Magnetic resonance imaging.

As illustrated in Figure 2, Models (i) and (ii) assigned points based on clinical variables and their corresponding linear predictive values. These models demonstrate that when considering a more comprehensive clinical scenario (such as combining MRI with high-confidence clinical diagnosis), the probability distribution of diagnosis significantly differs. Figure 2A presents the point allocation and corresponding linear predictive values for Model (i), showing the predicted diagnostic probability of Bell’s palsy at different point levels. It can be observed from the graph that as the linear predictive value increases, the probability of diagnosing Bell’s palsy also increases. Figure 2B displays the corresponding demonstration for Model (ii), where MRI results are considered as additional clinical variables. The disparities in point allocation and probability prediction between the two models highlight the potential value of MRI in enhancing diagnostic accuracy.

**Table 2:** Univariable associations between clinical predictors and Bell’s palsy diagnosis using high confidence clinical diagnosis with and without MRI

**Model (i) reference standard: high confidence (≥80%) clinical diagnosis**				
**Item variable**	**Patients with Bell’s palsy**	**Patients without Bell’s palsy**	**Univariable odds ratio**	**95% CI**
The facial crease and nasolabial fold disappear	143 (59.58)	97 (40.42)	4.66	2.23, 9.73
The forehead unfurrows	151 (62.92)	89 (37.08)	54.36	20.07, 157.25
Food and saliva pool in the affected side of mouth	147 (61.25)	93 (38.75)	11.67	5.24, 25.98
Facial sensation is preserved on the affected side	133 (55.42)	107 (44.58)	1.81	0.93, 3.53
Vesicular eruption in ear canal or pharynx without pronounced prodrome of pain	150 (62.5)	90 (37.5)	7.31	2.799, 19.14
Denial history of tick exposure, rash, or arthralgias	155 (64.58)	85 (35.42)	293.97	39.12, 2209.03
**Model (ii) reference standard: high confidence (≥80%) clinical diagnosis plus MRI**				
**Item variable**	**Patients with Bell’s palsy**	**Patients without Bell’s palsy**	**Univariable odds ratio**	**95% CI**
The facial crease and nasolabial fold disappear	128 (53.33)	112 (46.67)	4.25	1.99, 9.05
The forehead unfurrows	134 (55.83)	106 (44.17)	30.79	11.62, 81.60
Food and saliva pool in the affected side of mouth	131 (54.58)	109 (45.42)	9.07	3.99, 20.63
Facial sensation is preserved on the affected side	116 (48.33)	124 (51.67)	1.25	0.64, 2.42
Vesicular eruption in ear canal or pharynx without pronounced prodrome of pain	133 (55.42)	107 (44.58)	5.14	1.97, 13.39
Denial history of tick exposure, rash, or arthralgias	138 (57.5)	102 (42.5)	165.00	22.21, 1225.83

CI: Confidence intervals; MRI: Magnetic resonance imaging.

Through diagnostic modeling, we identified two potential categories, the first containing 104 patients and the second containing 95 patients. The overall agreement between the clinical diagnostic groups defined by Model (i) and the two latent categories was 82%, with a kappa coefficient of 0.71 (95% CI 0.63–0.79), depicting significant consistency [47]. This demonstrates the appropriateness of the clinical diagnostic reference standard. The overall agreement between the clinical diagnostic groups defined by Model (ii) and the two latent categories was 71%, with a kappa coefficient of 0.44 (95% CI 0.36–0.53), indicating a moderate level of consistency.

Following the univariate analysis, all predictive variables showed significant associations with the diagnostic results using both reference standards (*P* < 0.01), as shown in Table 2. The ORs for Model (i) were consistently higher compared to Model (ii). For the Model (i) diagnosis, the predictive variable most strongly associated was “denial of tick exposure history, rash, or joint pain,” with an exceptionally high OR of 293.97. Similarly, in Model (ii), the same variable exhibited the highest diagnostic relevance (OR ═ 165.00; 95% CI 39.12–2209.03).

In conclusion, this study identified multiple clinical predictive factors significantly associated with the diagnosis of Bell’s palsy through univariate analysis. Particularly, the variable of denying a history of tick exposure, rash, or joint pain demonstrated high diagnostic relevance under both the high-confidence clinical diagnosis alone and when combined with MRI results as diagnostic criteria.

### Multivariable diagnostic model analysis and prediction

In the multivariable analysis of 240 patients with Bell’s palsy, we developed two diagnostic models. The first model (Model i) was based on clinically diagnosed high confidence (≥ 80%), while the second model (Model ii) incorporated MRI confirmation in addition to the clinical diagnosis. The detailed results of these analyses are presented in Table 3. In the final established models, the indicators “facial folds and nasolabial grooves disappearance” and “preservation of sensation on the affected side of the face” were not included in both models, indicating their lack of statistical significance in the predictive models (*P* ≥ 0.05). On the other hand, the four other indicators—“smooth forehead,” “accumulation of food and saliva on the affected side of the mouth,” “sudden vesicle eruption in the ear canal or pharynx without apparent prodromal symptoms,” and “denial of tick exposure history, rash, or joint pain”—were retained in both models, highlighting their important predictive value in diagnosing Bell’s palsy.

**Table 3:** Multivariable associations between clinical assessment items and Bell’s palsy diagnosis in models with and without MRI confirmation

**Variable**	**Model (i)**		**Model (ii)**	
	**Beta**	**OR (95% CI)**	**Beta**	**OR (95% CI)**
The facial crease and nasolabial fold disappear	NS	NS	NS	NS
The forehead unfurrows	2.64	14.04 (3.74, 52.66)	1.90	6.69 (2.00, 22.34)
Food and saliva pool in the affected side of mouth	1.80	6.05 (1.58, 23.24)	1.16	3.19 (1.03, 9.83)
Facial sensation is preserved on the affected side	NS	NS	NS	NS
Vesicular eruption in ear canal or pharynx without pronounced prodrome of pain	2.36	10.63 (2.69, 41.98)	1.43	4.18 (1.24, 14.12)
Denial history of tick exposure, rash, or arthralgias	5.67	289.54 (34.68, 2417.24)	4.60	96.50 (12.47, 746.97)
Intercept	−9.43		−7.32	
AUC		0.962 (0.935, 0.989)		0.879 (0.835, 0.923)

Model (i): Confidence ≥ 80% Bell’s palsy clinical diagnosis; Model (ii): Confidence ≥ 80% Bell’s palsy clinical diagnosis plus confirmatory MRI findings. The predicted probability of Bell’s palsy can be calculated using the following formulas: Model (i): Probability (Bell’s palsy +) ═ 1/1 + exp −[−9.43 + (the forehead unfurrows × 2.64) + (food and saliva pool in the affected side of mouth × 1.80) + (vesicular eruption in ear canal or pharynx without pronounced prodrome of pain × 2.36) + (denial history of tick exposure, rash, or arthralgias × 5.67)]; Model (ii): Probability (Bell’s palsy +) ═ 1/1 + exp −[−7.32 + (the forehead unfurrows × 1.90) + (food and saliva pool in the affected side of mouth × 1.16) + (vesicular eruption in ear canal or pharynx without pronounced prodrome of pain × 1.43) + (denial history of tick exposure, rash, or arthralgias × 4.60)]. OR: Odds ratios; CI: Confidence intervals; AUC: Area under the receiver operating characteristic curve; NS: Non-significant at *P* ≥ 0.05.

The ORs in Model (i) were consistently higher than in Model (ii), suggesting that these features have a stronger diagnostic predictive ability for Bell’s palsy when MRI results are not considered. Among these, the OR for “denial of tick exposure history, rash, or joint pain” was the highest in Model (i), at 289.54 (95% CI 34.68–2417.24), demonstrating significant predictive value also in Model (ii) with an OR of 96.50 (95% CI 12.47–746.97).

**Figure 3.**
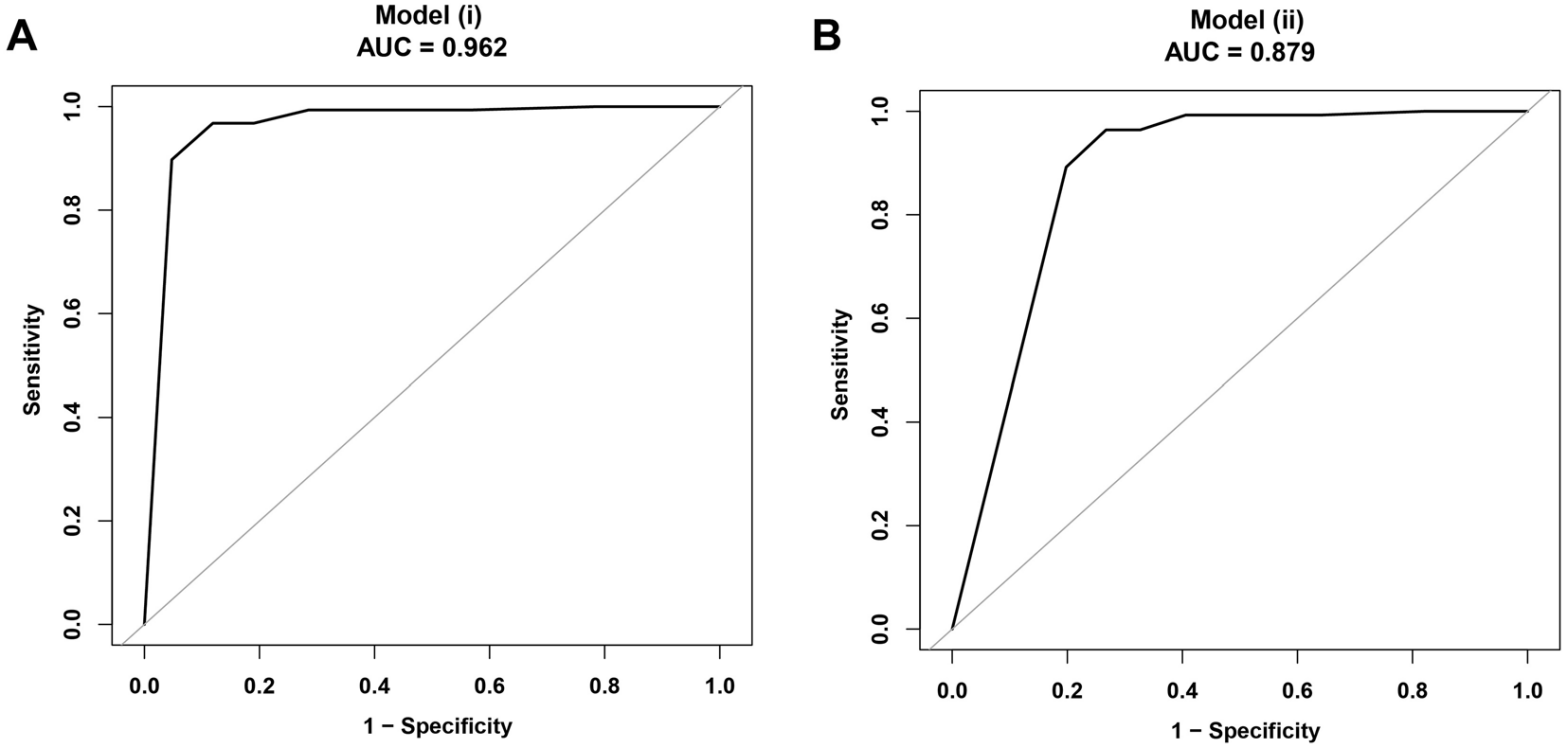
**ROC curve of the diagnosis model for Bell’s palsy.** (A) Showcasing the ROC curve and AUC value of Model (i), which predicts the diagnosis of Bell’s palsy based on highly confident clinical assessments; (B) Demonstrating the ROC curve and AUC value of Model (ii), which incorporates MRI results in addition to Model (i). Data from 240 Bell’s palsy patients were utilized for model development and validation. The ROC curve assesses the sensitivity and specificity of the model at various thresholds, while the AUC value reflects the overall diagnostic accuracy of the model. The 95% CI of the AUC value provides statistical significance regarding the model’s performance. Specific markers indicate the relationship between the model’s sensitivity (true positive rate) and 1-specificity (false-positive rate). ROC: Receiver operating characteristics; AUC: Area under the curve; MRI: Magnetic resonance imaging; CI: Confidence interval.

Through this study, we derived two highly accurate diagnostic models for Bell’s palsy, differing mainly in the inclusion of MRI confirmation results. The AUC values for the two models were 0.962 (95% CI 0.935–0.989) and 0.879 (95% CI 0.835–0.923), respectively (Figure 3), confirming the high effectiveness of these models in diagnosis. By utilizing these models, specific formulas can be employed to calculate the predictive probability of Bell’s palsy, offering a robust quantitative tool for clinical diagnosis.

In conclusion, this study successfully constructed two diagnostic prediction models for Bell’s palsy through multivariable analysis. It verified the importance of specific clinical indicators in diagnosing Bell’s palsy while also demonstrating the added value of MRI in enhancing diagnostic accuracy.

### Predictive model performance and scoring method for Bell’s palsy based on clinical assessment

**Figure 4.**
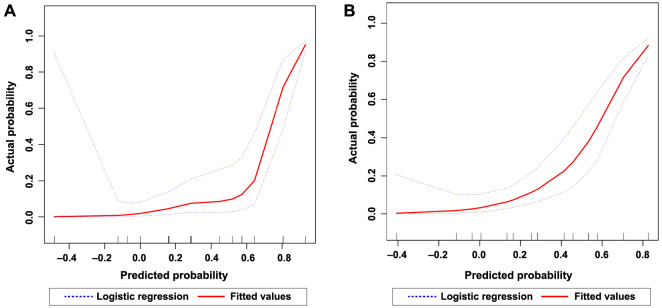
**Model calibration curve for the diagnosis of Bell’s palsy.** (A) Illustrating the calibration of Model (i). The solid red line in the graph represents a smooth estimate curve of the predicted probability of Bell’s palsy occurrence based on data from 240 patients, compared to the observed probability. Ideally, this curve should coincide with the blue-dashed line representing perfect prediction. The statistical significance was assessed using the Hosmer–Lemeshow test, with a χ^2^ value of 160.1 for Model (i), indicating a strong goodness of fit with a *P* value of less than 0.001. (B) Depicting the calibration curve for Model (ii), incorporating MRI results as an additional predictive variable to Model (i). Similarly, the solid red line illustrates the relationship between predicted and observed probabilities, with the calibration assessed by a χ^2^ value of 193.8 and a *P* value of less than 0.001, indicating a relatively poorer fit. MRI: Magnetic resonance imaging.

We subsequently compared the calibration performance of two predictive models for Bell’s palsy and their application in clinical practice. The slope shape in the calibration plot (Figure 4) indicates that Model (i) (Figure 4A) exhibits good calibration, whereas Model (ii) (Figure 4B) demonstrates relatively poor calibration. Utilizing the Hosmer–Lemeshow statistical test on the observed data, the results support the goodness of fit for Model (i) (χ2 ═ 160.1; *P* < 0.001), while Model (ii) shows inadequate calibration (χ2 ═ 193.8; *P* < 0.001).

In terms of discriminative ability, Model (i) performs close to perfection (AUC ═ 0.96, 95% CI 0.94–0.99), while Model (ii) shows excellent performance (AUC ═ 0.88, 95% CI 0.83–0.92). After adjustment through bootstrapping, there were no changes in the AUC values for both models. To better utilize the high-performing Model (i), we developed a simple scoring method by converting the β coefficient values into integers. The maximum total score is 13 (Table 4). The predicted probability corresponding to each total score was calculated. When employing this clinical diagnostic model (with high-confidence clinical diagnosis as the reference standard), a total score of 12 or higher indicates a high probability of Bell’s palsy diagnosis (at least 82%). Below this threshold, based on the ROC curve coordinates, the model exhibits a sensitivity of 0.90 and a specificity of 0.95.

**Table 4:** Scoring tool for predicting Bell’s palsy based on clinical assessment items in Model (i)

**Variable in the model**	**Does the patient:**										**Score**
The forehead unfurrows	Report the forehead disappearing on the affected side										3
Food and saliva pool in the affected side of mouth	Report food and saliva gathering on the affected side of mouth										2
Vesicular eruption in ear canal or pharynx without pronounced prodrome of pain	Report vesicular eruptions in the ear canal or pharynx without pronounced prodrome of pain										2
Denial history of tick exposure, rash, or arthralgias	Deny history of tick exposure, rash, or arthralgias										6
**Sum score**	**0**	**2**	**4**	**5**	**6**	**7**	**8**	**9**	**10**	**11**	**13**
*n*	3	15	19	7	5	12	12	1	5	17	144
Observed Bell’s palsy (%)	0	0	5	0	0	0	33	0	0	65	97
Mean predicted probability of Bell’s palsy (%)	**0**	**0**	**0**	**13**	**16**	**29**	**36**	**45**	**64**	**71**	**93**

In conclusion, this research provides an effective predictive tool for the clinical diagnosis of Bell’s palsy and introduces a user-friendly scoring method for clinical application. This scoring system is user-friendly, enabling healthcare providers to promptly assess the probability of a patient having Bell’s palsy, thus supporting more accurate diagnostic decisions.

### Sensitivity analysis of the MRI-based diagnosis model for Bell’s palsy

In a sensitivity analysis using MRI results as the sole reference standard, the model retained predictive variables including “forehead wrinkling” (OR 4.5, 95% CI 1.6, 12.4), “vesicular eruptions in the ear canal or pharynx without apparent prodromal symptoms” (OR ═ 1.4, 95% CI 0.4–4.3), and “denial of history of tick exposure, rash, or joint pain” (OR ═ 18.2, 95% CI 6.1–54.0), while “accumulation of food and saliva in the affected side of the mouth” did not enter the final model. The model exhibited modest discriminative ability with an AUC of 0.84 (95% CI 0.80–0.90).

Our analysis indicates that when MRI results serve as the reference standard, certain predictive factors remain crucial for diagnosing Bell’s palsy. Specifically, the predictor “denial of history of tick exposure, rash, or joint pain” demonstrated the highest OR, suggesting that careful medical history taking is essential for enhancing the model’s predictive performance in diagnosing Bell’s palsy. However, this model showed a lower AUC compared to others, implying that relying solely on MRI as the reference standard might limit diagnostic accuracy in the absence of additional clinical information.

## Discussion

Bell’s palsy is a common neurological disorder, and early diagnosis is crucial for patient recovery [33, 48]. In primary care settings, the diagnosis of Bell’s palsy is often challenging due to a lack of specialized neurologists [49]. Early diagnosis not only enhances treatment effectiveness but also reduces patients’ psychological and financial burdens [36, 50]. Therefore, the development of a simple and accurate diagnostic tool is imperative to improve patient management [51, 52]. This study aims to address this gap by constructing a precise identification model to enhance the diagnostic accuracy of Bell’s palsy in primary care environments.

Previous studies have predominantly focused on using traditional clinical assessment methods and neuroimaging tests for diagnosing Bell’s palsy [36, 53, 54]. While these methods are effective, they often rely on clinicians’ experience and judgment, introducing subjectivity [53, 55, 56]. Additionally, the high costs associated with neuroimaging tests may not always be feasible in primary care settings [57, 58]. In contrast to these studies, a notable feature of this research is the comparison of diagnostic models based on two reference standards. Both Model (i) and Model (ii) retain four crucial predictive factors considered key indicators in clinical practice for diagnosing Bell’s palsy [36, 59]. Specifically, the importance of “forehead wrinkling” as a significant clinical proxy distinguishing Bell’s palsy from central facial palsy has been reported in other diagnostic models as well [1, 2, 36, 60]. Furthermore, this study emphasizes the significance of denying tick exposure history, rash, or joint pain as predictive factors, as these characteristics are common in Lyme disease patients.

When carefully analyzing clinical assessment indicators, this study not only underscores the importance of “forehead wrinkling” and “denial of tick exposure history, rash, or joint pain” but also specifically mentions “food and saliva buildup in the affected side of the oral cavity” as a crucial indicator of facial nerve damage [20, 61]. Additionally, the association of vesicular eruptions in the ear canal or throat with pronounced preceding pain symptoms and their occurrence in conditions like acute and chronic otitis media, and Ramsay Hunt syndrome, are the focal points of this study [62, 63].

By comparing two models based on high-confidence clinical diagnosis and MRI results, this study demonstrates the significance of using different reference standards in diagnosing Bell’s palsy. Particularly, the nearly perfect calibration and discriminatory ability of Model (i) highlight the value of high-confidence clinical diagnosis as a reference standard. In contrast, while Model (ii) performs well in discriminatory ability, its calibration is poorer, indicating the need for further optimization when using MRI results as a reference standard. This comparison not only directs future research in exploring how to integrate clinical assessment with imaging results to enhance the overall performance of diagnostic tools but also underscores the importance of selecting the appropriate model in different clinical contexts.

Through cross-validation, this study confirms the minimal risk of overfitting in both models, indicating the robustness of the models. Particularly when using MRI as a reference standard, being able to predict various possible causes of facial nerve involvement is akin to previous studies utilizing MRI as a reference standard [64, 65]. Furthermore, the study’s findings accentuate the limitations in selecting different patient populations and reference standards and how these limitations impact the comparative abilities of diverse Bell’s palsy diagnostic models.

**Figure 5.**
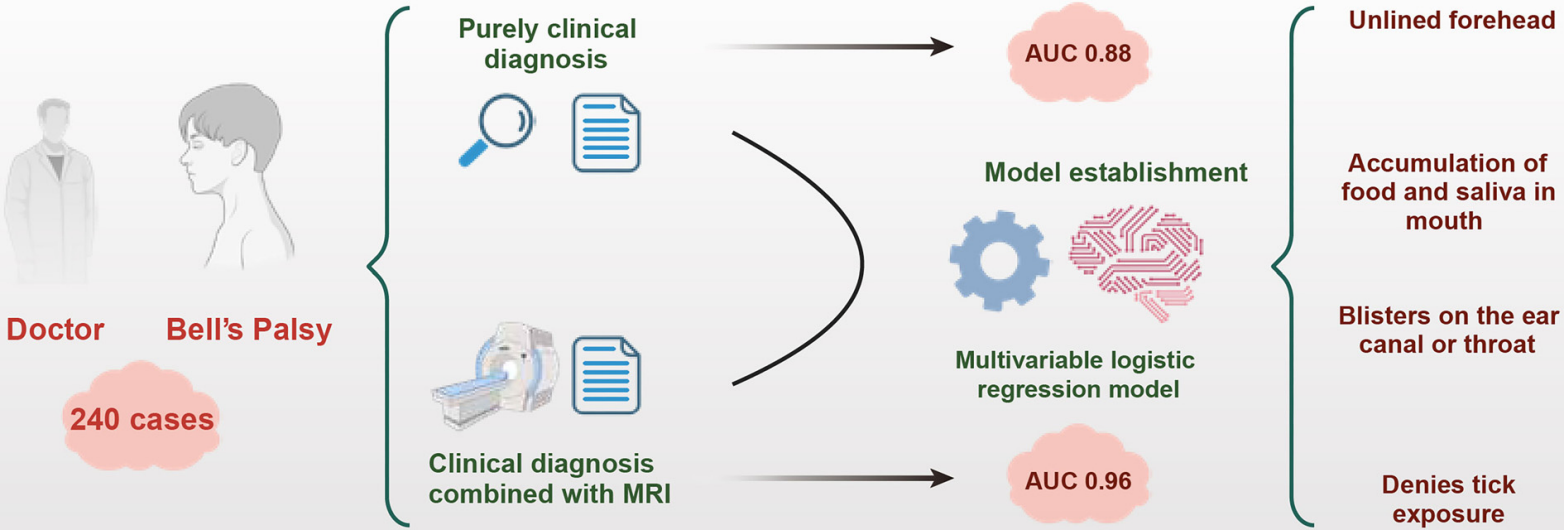
**Development and validation mechanism of the primary medical diagnosis model for Bell’s palsy in primary healthcare institutions.** AUC: Area under the curve; MRI: Magnetic resonance imaging.

From a clinical practice perspective, the models proposed in this study are user-friendly and particularly beneficial for primary care physicians who may need to make rapid decisions in the absence of readily available MRI resources. To aid in practical application, this study provides detailed guidance on the scoring system derived from our logistic regression model. Specific threshold scores that indicate a high likelihood of Bell’s palsy are defined, along with protocols for primary care physicians to follow upon obtaining a positive result [66]. However, the challenge lies in accurately applying these models to different patient populations and clinical scenarios. For instance, the diagnostic features of Lyme disease, such as rash and joint pain, may overlap with certain clinical presentations of Bell’s palsy, necessitating clinicians to consider these factors comprehensively when applying the models [1]. Moreover, certain indicators in the models, such as “forehead wrinkling” or “vesicular eruptions in the ear canal or throat” may manifest differently in diverse populations, requiring clinicians to be highly alert and adaptable.

Additionally, recognizing the variability in clinical skill levels and resource availability across different primary care settings, we discussed the potential challenges and limitations associated with the implementation of this model. Ongoing support and training for primary care teams are suggested to enhance the accuracy and reliability of the diagnostic process. These steps are aimed at preparing primary care providers for realistic scenarios they might encounter and offering solutions to overcome these barriers [20].

Future research should further delve into multiple directions for deeper exploration and expansion. Firstly, considering this study’s data is based on a specific population (240 patients with facial muscle weakness), future research should broaden the sample range, encompassing more diverse populations and geographical regions, to validate and optimize the model’s universality and accuracy. Secondly, further exploration and integration of novel clinical assessment indicators and imaging technologies could significantly enhance diagnostic model improvements. For example, leveraging recent advancements in artificial intelligence and machine learning technologies to develop new diagnostic models may enhance the diagnostic accuracy and efficiency of Bell’s palsy. Lastly, research should focus on the practicality and feasibility of applying these models in different clinical environments, especially in resource-constrained primary care settings, to drive the widespread clinical utilization of these models.

## Conclusion

This study utilized clinical assessment information to estimate the likelihood of Bell’s palsy occurrence in a primary healthcare setting (Figure 5). It explores, for the first time, the significant challenges, impacts, and inherent biases associated with selecting reference standards in diagnosing Bell’s palsy, by comparing models that use different reference standards. The study identified a set of key factors consistently associated with the recognition of Bell’s palsy: absence of forehead wrinkles, accumulation of food and saliva on the affected side of the mouth, vesicular eruptions in the ear canal or pharynx without preceding pain, denial of tick exposure, a history of rash or joint pain. Based on these findings, a simple scoring tool was developed, which may be particularly useful for clinical practitioners and researchers in determining whether a patient’s facial weakness is due to Bell’s palsy. This tool can assist in more accurately identifying homogeneous groups in a research setting.

## Data Availability

All data are available upon reasonable request.
